# Juvenile arthritis caused by a novel *FAMIN (LACC1)* mutation in two children with systemic and extended oligoarticular course

**DOI:** 10.1186/s12969-016-0124-2

**Published:** 2016-11-24

**Authors:** Tilmann Kallinich, Anne Thorwarth, Sae-Lim von Stuckrad, Angela Rösen-Wolff, Hella Luksch, Patrick Hundsdoerfer, Kirsten Minden, Peter Krawitz

**Affiliations:** 1 Charité University Medicine Berlin, Pediatric Pneumology and Immunology, Augustenburger Platz 1, 13353 Berlin, Germany; 2 Center for Chronically Sick Children of the Charité, Augustenburger Platz 1, 13353 Berlin, Germany; 3 Department of Pediatrics, University Clinic Carl Gustav Carus, TU Dresden, Fetscherstr. 74, 01037 Dresden, Germany; 4 Charité University Medicine Berlin, Pediatric Oncology and Hematology, Augustenburger Platz 1, 13353 Berlin, Germany; 5 Department of Rheumatology and Clinical Immunology, Charité University Medicine Berlin, Charitéplatz 1, 10117 Berlin, Germany; 6 Charité University Medicine Berlin, Institute of Medical Genetics and Human Genetics, Augustenburger Platz 1, 13353 Berlin, Germany

**Keywords:** Systemic juvenile idiopathic arthritis, Exome sequencing, *FAMIN, LACC1*

## Abstract

**Background:**

The pathophysiological origin of juvenile idiopathic arthritis (JIA) is largely unknown. However, individuals with presumably pathogenic mutations in *FAMIN* have been reported, associating this gene with a rare subtype of this disorder. *FAMIN*, that is formerly also referred to as *LACC1* or *C13orf31,* has recently been shown to play a crucial role in immune-metabolic functions and is involved in regulation of inflammasome activation and promotion of ROS production.

**Case presentation:**

We describe two siblings with severe familial forms of juvenile arthritis in which whole-exome-sequencing revealed a novel homozygous frameshift mutation (NM_153218.2:c.827delC¸. p.(T276fs*2) in *FAMIN*.

**Conclusions:**

The observation of a new deleterious mutation adds further evidence that pathogenic mutations in *FAMIN* are causal for a monogenic form of JIA. Furthermore the associated phenotype is not restricted to systemic JIA, but can also be found in other forms of familial juvenile arthritis.

## Background

Despite intensive analyses, the precise mechanisms leading to the phenotype of systemic juvenile idiopathic arthritis (sJIA) are still unknown (reviewed by [1]). Current understanding favors a model in which deregulation of innate immune mechanisms causes an upregulation of various soluble mediators, e.g. IL-1β [2], IL-18 [3], IL-6 [4] and the S100 molecules [5] and orchestrates a proinflammatory response reaction. Furthermore, an association of polymorphisms within the *IL1* and *IL1R* cluster genes [6, 7], the *macrophages migration inhibitor factor* (MIF) and the *IL10* cytokine cluster [8] has previously been described. Furthermore, a current genome wide association study (GWAS) identified the MHC locus as a bona fide susceptibility locus for the phenotype of sJIA [9].

In a previous report familial cases with sJIA have been linked to the gene *FAMIN*, (‘fatty acid metabolism – immunity nexus’*,* formerly also referred to as *LACC1* or *C13orf31*). Wakil et al. identified the homozygous missense mutation, p.Cys284Arg, in affected individuals from consanguineous Saudi Arabian families [10]. Furthermore, Arostegui, *et al.* reported a frame-shift mutation, p.Cys43Tyrfs*6, in three siblings from a consanguineous Moroccan family with severe highly inflammatory rheumatoid factor-negative polyarticular JIA [11].

These clinical reports coincide with a very recently published study that describe *FAMIN* as central regulator of endogenous fatty acid synthesis and their mitochondrial oxidation in macrophages [12]. In this respect, intact *FAMIN* is crucial in the production of inflammasome-mediated IL-1β as well as reactive oxygen species (ROS). *FAMIN* mutations are primarily described as having a loss-of-function effect on metabolic cell function and subsequent immunological response. Nevertheless, high dosages of lipopolysaccharides (LPS) induced catastrophic activation of IL-1β in *FAMIN* ^−/−^ mice, indicating that the profoundly impaired ‘energetic reserves’ might predipose these cells towards a pyroptotic, pro-death response.

We here present two siblings with severe forms of juvenile arthritis both harbouring a novel homozygous frameshift mutation within *FAMIN*. One sibling additionally developed precursor-B cell acute lymphoblastic leukemia (ALL). This report extends the phenotype of monogenic juvenile arthritis and adds evidence for the causative role of *FAMIN* in these cases.

## Case presentation

**Fig. 1:**
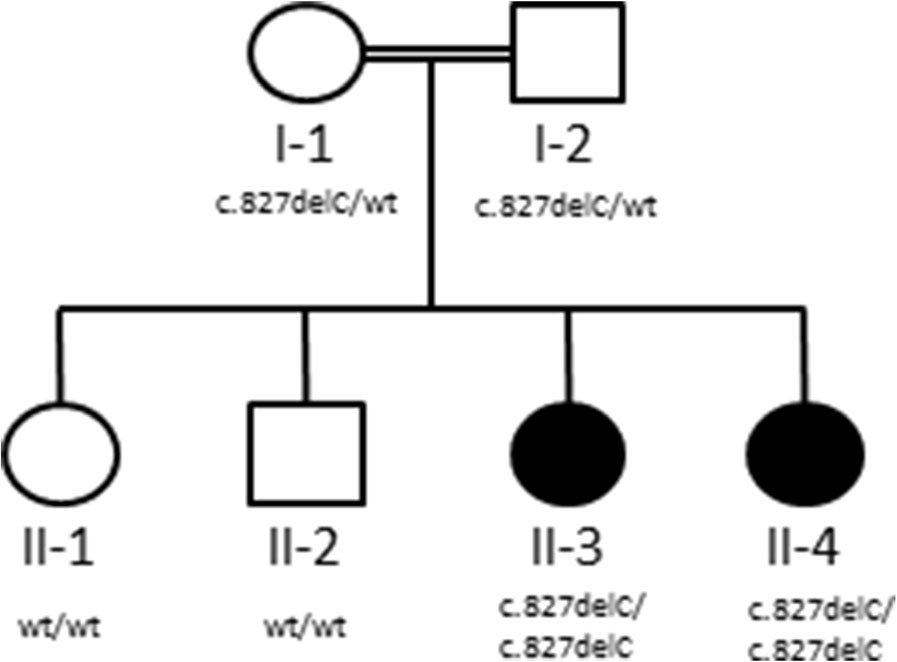
Pedigree

The consanguineous family originates from Lebanon (Fig. 1). The two affected girls as well as the two healthy siblings were born in Germany. Neither the parents nor the other siblings ever showed evidence of arthritis, systemic inflammation or recurrent infections.

At the age of 16 months the older child (II-3) developed simultaneously severe polyarthritis of wrists, knees and ankles, lymphadenopathy, extreme sensitivity to body contact, quotidian fever and remarkably elevated inflammatory markers (CRP 121 mg/l, leukocytes 18/nl). No serositis or exanthema was noticed. After intensive exclusion of other causes including bone marrow aspiration, systemic juvenile idiopathic arthritis was diagnosed according to the International League of Associations for Rheumatology (ILAR)-criteria.

The treatment consisted of repeated intraarticular steroid injections (shoulders, elbows, hands, hips, knees and ankles), non-steroidal anti-inflammatory drugs, high doses of corticosteroids (2 mg/kg) and methotrexate. Etanercept and Anakinra did not lead to a sufficient disease control. Finally, clinical remission was achieved 3,5 years after introduction of tocilizumab and methotrexate in addition to low dose steroids (0,2 mg/kg) and non-steroidal anti-inflammatory drugs.

At the age of six, the girl developed thrombocytopenia (56/nl) and precursor-B cell ALL was diagnosed. Over seven months the girl was treated with intensive chemotherapy according to the AIEOP-BFM ALL 2009 protocol. During this time she repeatedly suffered from severe flares of arthritis even shortly after chemotherapy (e.g. cyclophosphamide 1 g/m^2^), which resolved spontaneously.

At the age of eight, ALL relapse (bone marrow and central nervous system) was diagnosed. Second remission was achieved by chemotherapy according to the ALL-REZ BFM 2002 protocol. Allogeneic bone marrow transplantation from an unrelated 9/10 HLA-identical donor was performed after total body irradiation (12Gy + 6 Gy CNS boost) and high-dose chemotherapy. Post-transplant course was complicated by acute grade IV graft-versus-host disease of liver and skin. With respect to the ALL and the juvenile arthritis the girl is in continuous remission without immunosuppressive therapy since 24 months after bone marrow transplantation.

The seven-year-old female sister of the same family (II- 3) developed first manifestations of arthritis (knee and ankles) at the age of 15 months, which was initially classified as oligoarticular juvenile idiopathic arthritis and treated with intraarticular steroidinstillation and nonsteroidal anti-inflammatory drugs.

Eight months later the juvenile idiopathic arthritis flared up with a symmetric polyarthritis (shoulder, elbows, knees, ankles, wrists) and thus reclassified as extended oligoarticular JIA. Unusually high inflammatory makers (e.g. CRP 221 mg/l, leucocytes 18/nl) were noticed, but fever, rash, organomegaly or lymphadenopathy was not present. Treatment with methotrexate and systemic steroids (1 mg/kg) was started. Due to uncontrolled systemic inflammation adalimumab in combination with hydroxychloroquine was introduced and sufficient control of the inflammation was achieved.

In both children whole-exome-sequencing revealed the homozygous one base pair deletion in *FAMIN* NM_153218.2:c.827delC. The mutation results in a frameshift and a premature stop codon, p.(Thr276fs*2). Both parents are heterozygous carriers of the deletion and the two unaffected children showed the wildtype of the gene.

## Conclusion

In this report we describe the novel homozygous frameshift mutation, p.Thr276fs*2, in *FAMIN*. The observation that a frameshift mutation leads to a similar juvenile arthritis phenotype comparable to the previously described mutations strongly supports the significance of these gene variants in the development of juvenile arthritis. Our cases demonstrate, that *FAMIN* mutations can also be found in extended oligoarticular juvenile arthritis associated with systemic inflammation.

Interestingly, all case reports with deleterious mutations in *FAMIN* have so far been from families of Arab ethnicity. However, this might simply be due to the fact, that the identification of pathogenic mutations is facilitated in highly consanguineous families. It is noteworthy, that none of the reported pathogenic mutations has been observed in the ExAC cohort.

Previously, genome wide association studies (GWAS) of unrelated patients with sJIA did not reveal significant -log_10_ p values for the *FAMIN* locus [9]. Therefore, variants within this gene do not seem to play a role in patients suffering from the common forms of sJIA. However, GWA studies can only detect frequent variants. Cases of juvenile arthritis caused by *FAMIN* mutations have to be distinguished from the common forms of (s)JIA. Since these cases are not restricted to systemic forms and are not ‘idiopathic’ they should be referred to as *monogenic juvenile arthritis*. The severity of the phenotypes in terms of difficult disease control underlines the clinical need to separate these entities from common forms of sJIA. Whether the phenotype of monogenic juvenile arthritis is restricted to familial cases has to be analyzed in larger cohort studies.

Large GWAS, including several thousands of patients with infectious and autoimmune diseases give evidence to a major role of *FAMIN* in the inflammatory response: in patients with leprosy an association of the common variant p.Ile254Val (rs37641479) located within *FAMIN* was found and the gene activity is discussed as a host-resistance factor in leprosy [13–16]. The same variant was found in cohorts with increased susceptibility to Crohn’s disease pointing to a common host response pathway in leprosy and Crohn’s disease [17, 18]. Furthermore, the ‘familial sJIA variant’ p.Cys284Arg was detected in a family with early-onset Crohn’s disease [19]. Crohn’s disease and infections share some phenotypical aspects of JIA, e.g. the occurrence of arthritis and the systemic inflammatory reaction. Current pathophysiological concepts of JIA furthermore indicate an important role of a dysregulation within pathogen-sensing mechanisms, e.g. the inflammasomes. Together with the observation of similar genetic alterations this suggests that pathophysiological pathways are shared in these three diseases. Moreover, the now known function of *FAMIN* and the described variants causing different forms of JIA these underlines that the found variants are not simply in tight linkage disequilibrium with a causative variant but are truly causative.

When introduced into a murine model, the variants p.Cys284Arg, associated with ‘familial sJIA’ and early onset Crohn’s disease, as well as the SNP p.Ile254Val associated with increased susceptibility to Crohn’s disease and leprosy, suppressed the observed metabolic changes as measured by means of extracellular acidification rate and oxygen-consumption rate [12]. Thus, an *ex vivo* assay exists to determine the gene-dose effect on metabolic cell function.

The altered metabolic cell function observed *ex vivo,* reflects the severity of clinical phenotypes. In this respect, p.Ile254Val led to rather mild cellular changes with increased susceptibility to leprosy and Crohn’s disease, whereas the p.Cys284Arg variant caused stronger metabolic alterations resulting in a more severe phenotype with early-onset Crohn’s disease or ‘familial sJIA’, respectively. This genotype-phenotype correlation is furthermore supported as the novel frameshift mutation p.Thr276fs reported here is of higher predicted pathogenicity than the missense variant p.Cys284Arg. This hypothesis is supported by the fact, that the stop codon at amino acid position 276 is located upstream of 284 and therefore affects the consecutive N-terminal amino acids 288 to 430.

After BMT, the child (II-3) does not show any signs of arthritis. Given the fact, that *FAMIN* is closely linked to the inflammatory function of macrophages, this observation underlines the pathophysiological importance of this cell type in sJIA.

Taken together, we present a family with closely related parents with two children suffering of severe forms of monogenic juvenile arthritis caused by homozygous variants within *FAMIN*. In line with the previously described cases of *FAMIN*-related familial systemic and polyarticular JIA, the reported association with other inflammatory and infectious diseases, respectively, as well as the currently published pathophysiological role of *FAMIN*, these cases add evidence for a causative role of the alterations in the development of these inflammatory phenotypes.

## Abbreviations

BMT: Bone marrow transplantation
GWAS: Genome wide association study
JIA: Juvenile idiopathic arthritis
LPS: Lipopolysaccharides
MIF: Migration inhibitor factor
ROS: Reactive oxygen species
sJIA: Systemic juvenile idiopathic arthritis
